# Comparison of digital PCR platforms and semi-nested qPCR as a tool to determine the size of the HIV reservoir

**DOI:** 10.1038/srep13811

**Published:** 2015-09-09

**Authors:** K. J. Bosman, M. Nijhuis, P. M. van Ham, A. M. J. Wensing, K. Vervisch, L. Vandekerckhove, W. De Spiegelaere

**Affiliations:** 1 Department of Medical Microbiology, Virology, University Medical Centre Utrecht, The Netherlands; 2 Department of Internal Medicine, HIV Translational Research Unit, University Ghent & University Hospital Ghent, Belgium.

## Abstract

HIV persists in latently infected cells of patients on antiretroviral therapy (ART). This persistent proviral DNA reservoir is an important predictor of viral rebound upon therapy failure or interruption and forms a major obstacle towards cure. Accurate quantification of the low levels of persisting HIV DNA may aid patient monitoring and cure research. Digital PCR is a promising tool that enables direct absolute quantification with high sensitivity. With recent technological advances, several platforms are available to implement digital PCR in a clinical setting. Here, we compared two digital PCR platforms, the Quantstudio 3D (Life Technologies) and the QX100 (Bio-Rad) with a semi-nested qPCR on serial HIV DNA dilutions and DNA isolated from PBMCs of ART-suppressed patients. All three methods were able to detect target to the lowest levels of 2.5 HIV DNA copies. The QX100 excelled in having the least bias and highest precision, efficiency and quantitative linearity. Patient sample quantifications by the QX100 and semi-nested qPCR were highly agreeable by Bland-Altman analysis (0.01 ± 0.32 log_10_). Due to the observation of false-positive signals with current digital PCR platforms however, semi-nested qPCR may still be preferred in a setup of low quantity detection to discriminate between presence or absence of HIV DNA.

Current treatment of HIV depends on the use of antiretroviral drugs that block different steps in the HIV replication cycle. To verify that ART is successfully suppressing HIV replication, patients are routinely monitored for the abundance of HIV particles in their bloodstream by quantification of viral RNA in plasma. However, despite effective viral suppression in plasma, the virus remains present in a so-called latent reservoir of infected cells harboring replication competent proviral HIV DNA in their genome^1^,^2^. Consequently, HIV RNA quantification in plasma is inadequate to monitor the size and dynamics of the latent HIV DNA reservoir.

Total cellular HIV DNA has been proposed as an alternative marker to measure the size of the latent reservoir. The predictive role of HIV DNA as a marker of the replication competent reservoir is debatable because most HIV DNA is replication deficient^3^. However, accumulating clinical data indicate that HIV DNA can be used as a marker in cure research and patient monitoring to predict remission of HIV after treatment with ART or other therapies directed at eradicating the latent reservoir. The size of the HIV DNA reservoir was shown to correlate with the time to viral rebound^4^,^5^ and the viraemia setpoint^6^ upon ART interruption. In agreement with these findings, a recent clinical trial in humans found that reductions in HIV DNA levels were associated with durable viral suppression after ART treatment interruption following a pegylated interferon alfa-2a stimulation^7^. In addition, HIV DNA levels are predictive of disease progression in untreated patients^8^,^9^, even more so than plasma viral RNA load^5^. Together, these findings indicate the importance of HIV DNA quantification as a marker for HIV disease and persistence research.

Because of the low frequency of proviral HIV DNA in immune cells from the peripheral blood and tissue compartments, future clinical management and cure research will benefit from a system that can reliably quantify HIV DNA in patients. The system should specifically be able to detect low copy numbers as often observed in long-term ART-suppressed patients^3^ and in patients who underwent stem-cell transplantation in an attempt to clear the viral reservoir^10^. To date, most quantification techniques make use of real-time quantitative PCR (qPCR) to detect and quantify HIV DNA. However, interpretation of the resulting relative quantification to absolute quantities requires a standard dilution. This may introduce quantitative bias when PCR efficiencies are variable between samples. Variable PCR efficiencies are likely to occur in HIV-infected patients, as variations in the target sequence that lead to mismatches in the primer and/or probe sequence can affect PCR efficiency. Moreover, qPCR inaccuracy increases with lower target abundance due to the logarithmic nature of PCR^11^, thereby potentially complicating reliable quantification of the lowly abundant HIV DNA found in ART-suppressed patients.

The recent development of digital PCR (dPCR) platforms is a promising step forward, as this technology provides absolute quantification of DNA without the need of a standard curve and with a better tolerance to PCR inhibitors compared to qPCR^12^. Instead of measuring the real-time increase of fluorescence intensity within one sample, dPCR measures the end-point fluorescence of a large number of PCR partitions of the same sample in the limiting dilution range. By applying Poisson-statistics, the fraction of positive partitions is used as a measure for the number of targets in the sample, allowing direct absolute quantification^13^.

Although the concept of dPCR has been around for several decades, technological advances are now enabling a broad use of this technology^13^,^14^,^15^,^16^,^17^. The Droplet Digital™ PCR system (Bio-Rad) and the RainDrop^®^ Digital PCR system (RainDance Technologies) use microfluidics based droplet in oil solutions to make PCR partitions^14^,^18^,^19^. The QuantStudio^®^ 3D Digital PCR system (Life Technologies) divides the PCR partitions in a grid of wells, after which the PCR reaction takes place and the microchip is interpreted in a separate read-out unit^20^,^21^,^22^,^23^. DPCR has previously been described to provide absolute quantification of HIV with a sensitivity similar to or better than qPCR as shown in studies that assessed HIV RNA^24^, cell-associated HIV RNA^25^, total HIV DNA^26^,^27^,^28^,^29^ and HIV DNA 2-LTR circles^26^,^29^,^30^.

In the present study, we compared two differently operating dPCR platforms with a highly sensitive semi-nested qPCR for detecting lowly abundant HIV DNA copies. We used an identical primer-probe set for the most direct comparison between Bio-Rad’s QX100™ Droplet Digital™ PCR System, Life Technologies™ QuantStudio^®^ 3D Digital PCR system and a semi-nested qPCR assay. HIV DNA copy abundance was measured in both an artificial dilution series and in HIV-infected patient samples.

## Materials and Methods

### Patient samples

Ethical approval was obtained from the Ethics Committees of the University Hospital Ghent (ref. nr. B670201111928), all participants provided written informed consent and all experiments were performed in accordance with the approved guidelines and regulations. To assess performance on patient-derived samples, the abundance of total cellular HIV DNA was measured with all three methods in 20 ART-suppressed patients with undetectable plasma loads (<40 copies/mL). Peripheral blood mononuclear cells (PBMCs) were isolated from fresh blood by Ficoll gradient centrifugation and stored as dry pellets at −80 °C until further processing. As negative template control samples, water NTCs (no template controls) were used and PBMCs from HIV-negative healthy donors as PBMC DNA NTCs.

### DNA extraction and digestion

Total DNA was extracted from 5–10 × 10^6^ PBMCs or U1 cells using the DNeasy^**®**^ mini kit (Qiagen, Venlo, The Netherlands) using the standard protocol with an elution buffer (45 μl per sample) preheated at 56 °C for 5 min to increase the yield of the isolation procedure.

### U1 dilution series

In the first part of this study, a U1 cell line was diluted to construct a dilution series ranging from 160 to 2.5 HIV DNA copies per sample. The U1 cell line^31^,^32^, containing two HXB2/Lai HIV provirus integrations per cell^33^, was obtained from the NIH AIDS Reagent Program, Division of AIDS, NIAID. The cells were cultured in RPMI1640 with L-glutamine (Lonza), 10% Fetal Calf Serum (FCS, Sigma-Aldrich) and 10 μg/ml gentamicin (Invitrogen)^32^. After isolation, the DNA yield was measured by Qubit and diluted to contain 160 copies per 1.6 μl (0.264 ng/μl). This sample was used to create a two-fold dilution of samples containing 160, 80, 40, 20, 10, 5 and 2.5 HIV copies per 0.9 μl. Cell numbers were deduced from the amount of DNA, assuming a mean molecular weight of 615 Dalton or 1.023E-9 pg per nucleotide pair and a human genome length of 3234 Mb, the genome of one diploid U1 cell weighs 6.6 pg. As the U1 cell line carries two proviral HIV copies, 3.3 pg of U1 DNA should on average contain one HIV copy^34^. All samples were supplemented with HIV-negative donor PBMC DNA to standardize the level of total cellular DNA in all dilutions by supplementing to 1000 ng of donor PBMC DNA (the equivalent of ~150.000 cells) to a final concentration of the desired input copy numbers per 1.6 μl. Part of every dilution was digested with EcoRI (Roche Diagnostics) to prepare the samples for dPCR. Restriction digestion was performed by adding 0.2 μl of the EcoRI enzyme (10 U/μl) and 0.2 μl 10x buffer H to every 1.6 μl of sample. The undigested part of the dilution series was supplemented with a volume of water equal to that of the digestion mix, thereby also diluting the sample 1.25 times to ultimately contain the desired input copy numbers per 2 μl for measurement in the qPCR. The samples were measured in triplicate by each of the three methods (a total of 9 samples measured per dilution).

### Primers and probes

The same primer-probe set was used for all platforms. These primers amplify a part of the HIV genome that codes for capsid p24 using the gag1 assay. Gag_forward (5′-TGGGTAAAAGTAGTAGAAGAGAAGGCTTT-3′) was used as the forward primer and Gag_reverse (5′-CCCCCCACTGTGTTTAGCAT-3′) as the reverse primer. The gag1 assay uses the Taqman minor groove binding (MGB) FAM-probe Gag_MGB (5′-TCAGCATTATCAGAAGGAG-3′), which maps in between the two gag1 primers. The assay was only semi-nested in the real-time PCR, using the Gag_forward primer and a reverse primer located 3′ of the Gag_reverse primer, named 3′MA-1 (5′-GCTATGTCACTTCCCCTTGGTTCT-3′). As the primers were designed for subtype B, none of the primers have any mismatches with the HXB2 reference genome that was used to make the U1 cell line. Mismatches were found with the three subtype B reference genomes used in the HIV LANL subtype reference alignment (http://www.hiv.lanl.gov/content/sequence/QUICK_ALIGNv2/QuickAlign.html): B.NL.00.671_00T36.AY423387, B.TH.90.BK132.AY173951 and B.US.98.1058_11.AY331295. The Gag_forward primer has one mismatch in each of the three subtype B reference genomes, the Gag_MGB FAM-probe and the 3′MA-1 primer have one mismatch in one of the three reference genomes, and the Gag_reverse primer has no mismatches to any of the three reference genomes.

### QX100 dPCR

The reaction mix used to quantify in the QX100™ Droplet Digital™ PCR System (Bio-Rad), further referred to as the QX100, consisted of 10 μl 2x ddPCR™ super mix for probes (Bio-Rad), 800 nM primers, 300 nM probe and 2 μl of the restriction digest, into a final volume of 20 μl. Droplets were generated in the droplet generator (Bio-Rad) and PCR was performed in a T100™ thermal cycler (Bio-Rad). PCR amplification procedure consisted of an initial denaturation at 95 °C for 5 min, and 39 cycles of denaturation at 95 °C for 30 sec and annealing/elongation at 58 °C for 1 min. After PCR, read-out of positive versus negative droplets was performed with the droplet reader.

### Quantstudio 3D dPCR

The samples that were measured by the™ QuantStudio^®^ 3D Digital PCR System (Life Technologies), further referred to as the Quantstudio, were loaded onto the chips using the QuantStudio^®^ 3D Digital PCR Chip Loader in a mixture consisting of 2x Quantstudio^®^ 3D digital PCR mastermix, 300 nM of Gag_foward primer, 300 nM of Gag_reverse primer and 300 nM of Gag_MGB probe. The chips were sealed and loaded onto a GeneAMP^®^ PCR system 9700 (Applied Biosystems^®^) and cycled according to the following parameters: 96 °C for 10 minutes, followed by 39 cycles of 60 °C for 2 min and 98 °C for 30 sec, and a final extension at 60 °C for 2 min. After cycling, the end-point fluorescence of the partitions on the chips was measured by transferring the chips to the measurement unit (application version 1.1.3, algorithm version 0.13).

### Semi-nested real-time PCR

The real-time PCR uses two rounds of PCR amplification of the gag-region of p24. The first PCR is performed in a T100™ PCR machine (Bio-Rad) in a 25 μl mix containing 2 μl of DNA sample, 2.5 μl 10x home-brewed OT buffer (670 mM TrisHCl, 170 mM (NH_4_)_2_SO_4_, 10 mM β-Mercaptoethanol, 60 μM EDTA, 2 mg/mL BSA), 200 nM dNTPs, 2 mM MgCl_2_, 0.65 units DreamTaq polymerase (Promega) and 200 nM Gag_forward and 200 nM 3′MA-1 primers. The cycling parameters were: 94 °C for 3 min, followed by 15 cycles of 94 °C for 30 sec, 55 °C for 30 seconds and 72 °C for 1 min, and finally 72 °C for 5 min. The nested PCR was then performed on an Applied Biosystems® StepOnePlus™ Real-Time PCR system (which we will now call qPCR) with 5 μl of the first PCR as the template for the nested mix containing 12.5 μl of 2x Taqman mix, 300 nM of Gag_forward primer, 300 nM of Gag_reverse primer and 300 nM of Gag_MGB probe. The real-time cycling parameters were 50 °C for 2 min, 95 °C for 10 min, followed by 40 cycles of 95 °C for 15 sec and 60 °C for 1 min.

### Data analysis

Raw data analysis was performed with the specific software for each of the three platforms, i.e. QuantaSoft (version 1.3.2.0) for the QX100, Quantstudio 3D Analysis Suite Cloud Software v1.0 for the Quantstudio (https://apps.lifetechnologies.com/quantstudio3d) and StepOne Software v2.3 for qPCR. For the QX100 a common threshold was set at a fixed fluorescence intensity of 3000 based on the PBMC DNA NTCs and this threshold was used to assess all QX100 partitions. For the Quantstudio quantifications, automated threshold setting proved highly unreliable for the smallest input samples, and the analysis software does not enable direct comparison of the samples, thereby creating user bias when manually setting one uniform threshold. For this study, the threshold was user-defined at 4000. For qPCR, the Cq threshold was manually set at 0.025 for all samples of the dilution series experiment and at 0.029 for all samples of the patient material comparison. Bias was defined as the difference (in percent) of observed quantity from the expected quantity.

To compare precision between the three systems, we calculated the coefficient of variation (CV), which is the proportion of the standard deviation in relation to the mean, expressed as a percentage. The CV provides a rough estimation of the capability of the systems to produce reproducible quantities for three replicates that should contain the same number of targets. To be able to calculate CVs for the lowest input range, we replaced the results of replicates for which no target could be detected with 40 in case of qPCR and with 0.01 in case of digital PCR, but it should be noted that these undetermined results unrightfully increase the CV whereas an undetermined result may in fact be due to sampling error.

For the semi-nested qPCR, a transformation of the raw Cq data was performed to allow direct comparison of CV between the logarithmical output of the semi-nested qPCR and the linear dPCR output^11^. The Cqs were transformed to relative quantities by normalizing each Cq against the average Cq of the highest input sample (∆Cq) and using the empirically defined efficiency to calculate relative quantities using the formula q = 1/E^∆Cq^ (with q the relative quantity and E the PCR efficiency).

Quantitative linearity and efficiency of the measurements in the dilution series was assessed using robust regression analysis. Simple linear regression greatly suffers from outliers that may pull the regression towards their coordinates, whereas robust regression analysis is less susceptible to single outliers and provides a more efficient estimation of the slope of a dilution curve^35^. Robust regression was analyzed by using the biweight MM estimation which is an estimator that was demonstrated to be less influenced by outliers in the extremes of the standard curves^35^. Robust regression analysis was performed in R, using the lmRob package.

Agreement between the different methods for quantifying the same patient samples was assessed by Bland-Altman analysis. Bland-Altman analysis compares every observation of two methods by plotting the difference between observations against their average value. In the low ranges of the dilution series, errors of random sampling can increase variation between technical replicates^36^. To analyze the results without interference of the sampling error, all patient samples for which no target could be detected in one or more replicates by any method were excluded. Bland-Altman analysis was performed in R using custom scripts.Ethical approval was obtained from the Ethics Committees of the University Hospital Ghent (ref. nr. B670201111928), all participants provided written informed consent and all experiments were performed in accordance with the approved guidelines and regulations.

## Results

### Detection of HIV DNA in the U1 dilution series

All three methods were able to detect dilutions with high concentrations of HIV DNA (Table 1). The semi-nested qPCR only delivered a detectable signal for one replicate of the 5-copy dilution and for two replicates of the 2.5 copy sample. Semi-nested qPCR was completely negative in the PBMC DNA NTCs and in water NTCs. The QX100 was negative for one replicate in the 2.5 copy dilution and for all three replicates of the PBMC DNA NTCs (Table 1). The Quantstudio detected input in every single replicate of the dilution series, including the PBMC DNA NTCs and water NTCs (Supplementary Table S1).

In terms of precision (variation between technical replicates), the QX100 and the Quantstudio performed comparably; neither of the methods had a consistently higher or lower coefficient of variation (CV). A comparison of the CVs from the relative qPCR quantities with the output of the dPCR platforms revealed that the qPCR was less accurate compared to the QX100 and the Quantstudio (Table 1).

**Figure 1:**
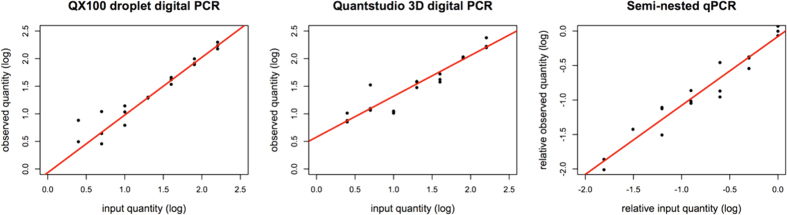
Expected and observed values of the dilution series. Input quantity plotted against the observed quantities of the dPCR platforms and against the Cq-values of the qPCR. Robust regression analysis reveals a quantitative efficiency of 104.5% for the QX100, 74% for the Quantstudio and 93.64% for the qPCR. Quantitative linearity is R^2^ = 0.85 for the QX100, R^2^ = 0.78 for the Quantstudio and R^2^ = 0.83 for the qPCR.

Both dPCR platforms overestimated the expected dilution series quantities (bias 8.1% for the QX100 and 28.8% for the Quantstudio) (Fig. 1). The observed quantities from the QX100 were closer to the input quantities compared to the Quantstudio for every step in the dilution series (Table 1). The Quantstudio overestimated the two dilutions with the least input DNA most severely, possibly due to the false-positive reactions that were also observed in the PBMC DNA NTCs and water NTCs (Supplementary Table S1). The semi-nested qPCR could not be assessed for bias because its absolute quantification depends on a relative quantification based on the standard itself.

Quantification efficiency (E) as derived from the slope of the robust regression trend line was E = 74%, 93.6% and 104.5% with the Quantstudio, the semi-nested qPCR and the QX100 respectively (Fig. 1). Omitting undetermined values, the R^2^ amounted to 0.78 for the Quantstudio, 0.83 for the qPCR and 0.85 for the QX100.

### Detection of HIV DNA in patient samples

The detectability of HIV DNA copies in HIV-infected ART-suppressed patient samples differed among the three methods. The semi-nested qPCR detected HIV DNA in at least one of the triplicates of 15 out of 20 patients with a detectability of 62% across all triplicates (Supplementary Table S2). The QX100 detected target in at least one of the triplicates in all patients with an 80% detectability of all triplicates. The Quantstudio detected HIV DNA in all patients and did not return an undetermined result in any of the replicates.

**Figure 2:**
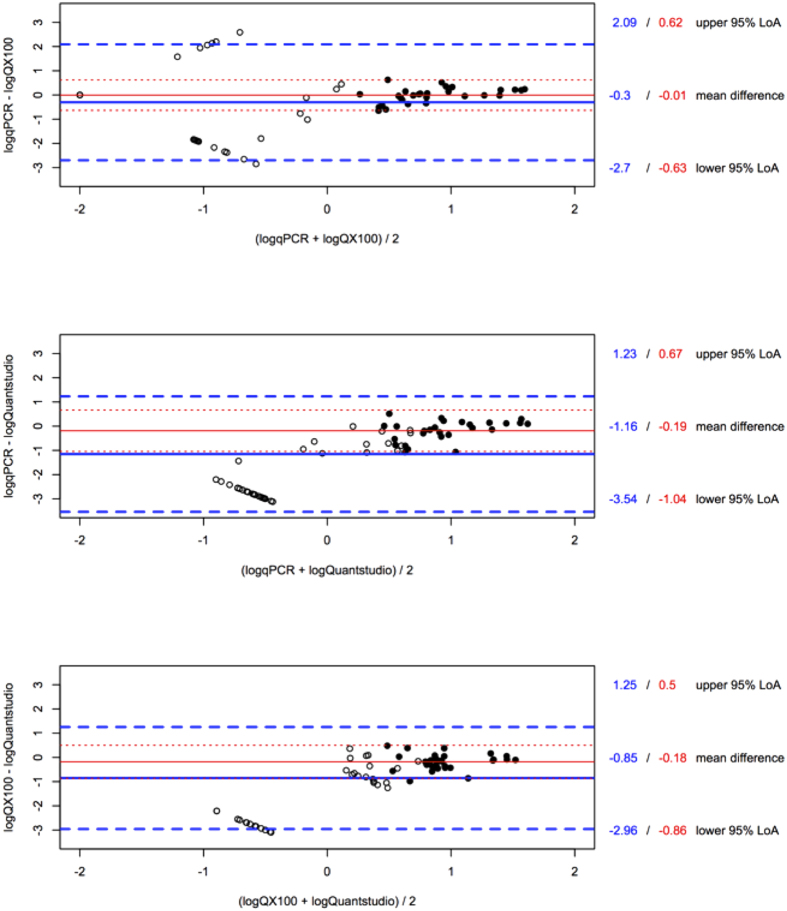
Bland-Altman plots of HIV DNA quantification in patient-derived PBMC DNA. Comparing qPCR with QX100, qPCR with Quantstudio and QX100 with Quantstudio with all data (filled and open circles, bold blue lines) and using only samples with three detectable replicates in all methods (filled circles, red lines). Lines indicating mean difference are solid, lines indicating LoA’s are dashed.

As can be inferred from the Bland-Altman analysis, the semi-nested qPCR and the QX100 proved most similar: −0.30 ± 1.22 log10 (95% Limits of Agreement (LoA) of −2.70 to 2.09 log10), whereas agreement between the QX100 and the Quantstudio was found to be −0.85 ± 1.07 log10 (LoA −2.96 to 1.25 log10). The semi-nested qPCR and the Quantstudio had the highest average difference with an agreement of −1.16 ± 1.22 log10 (LoA −3.54 to 1.23 log10) (Fig. 2). This initial Bland-Altman analysis revealed that both the agreement and LoA levels were biased because of the samples with low HIV DNA returning one or more undetectable replicates. The actual concentration of HIV DNA per replicate reaction in these samples is close to or less than one. In these low ranges, the stochastic sampling error causes substantial variation between replicates, which hampers the comparison of different methods^36^. This was statistically confirmed by first regressing the differences between methods on the averages and then regressing the absolute values of the residuals on the average between both methods. The latter regression was significant, indicating a non-constant variance between both methods (Supplementary Figure S1 A–F).

The analysis on a subset of 9 patient samples for which target could be detected in all replicates increased the agreement among the three methods (Fig. 2). The semi-nested qPCR and the QX100 had the best agreement, being −0.01 ± 0.32 log_10_ (LoA −0.63 to 0.62 log_10_) and the QX100 and the Quantstudio comparison agreed −0.18 ± 0.35 log_10_ (LoA −0.86 to 0.50 log_10_). The qPCR and the Quantstudio were again the least similar methods with an average agreement of −0.19 ± 0.44 log_10_ (LoA −1.04 to 0.67 log_10_). Regression of the absolute values of the residuals on the average between both methods was no longer significant in the limited dataset, indicating a constant variance between both methods in this range (Supplementary Figure S1 G–I).

## Discussion

Owing to recent technological developments, dPCR is becoming a highly investigated method for absolute quantification of DNA molecules. Although its current implementations do not match the full theoretical potential^26^,^37^,^38^, dPCR is theoretically able to deliver direct absolute quantification of DNA copies provided a random distribution of samples in equally sized partitions^13^,^14^, and recent data show that dPCR is more accurate compared to classical qPCR^39^,^40^,^41^. Here, we evaluated two dPCR platforms with a highly sensitive semi-nested qPCR method to quantify HIV DNA in samples with low target abundance. Our data show that dPCR can accurately quantify low levels of HIV DNA in patient samples, but that semi-nested qPCR may still be preferred for discriminating true positive from false-positive reactions.

The qPCR was the least precise of the three systems with a higher median CV than the QX100 and the Quantstudio in both the dilution series (Table 1) and the patient samples (Supplementary Table S3). An explanation for this may be found in the nested approach of the qPCR. This approach involves more manual handling, rendering it more susceptible to technical bias. In addition, the logarithmic measurement of target abundance performed by qPCR is known to inherently decrease accuracy and precision^11^,^42^. The observation that the QX100 has the best efficiency E = 104.5% and the highest adherence to the robust regression trend line R^2^ = 0.88 indicates that the QX100 produces the most reliable output (Fig. 1). The low efficiency E = 74% of the Quantstudio may be explained by the overestimation of low input samples, caused by the presence of false-positive partitions. False-positive detection proved to be a severe problem in the Quantstudio. The platform gave positive partitions in both of the PBMC DNA NTCs and all of the 8 water NTCs that were tested. None of the PBMC DNA NTCs tested by the QX100 or the semi-nested qPCR returned an output, but false-positives were observed in the water NTCs tested by the QX100 (Supplementary Table S1). This observation is in accordance with other published data^11^,^25^,^26^,^43^,^44^ and indicates that the presence of false-positive partitions in the samples cannot be excluded with the QX100 either.

Both dPCR platforms overestimated the amount of input DNA, but the bias between the expected and observed quantities of the QX100 was substantially lower (median 8.1%) compared to that of the Quantstudio (median 28.8%) (Table 1). Bias in the dPCR platforms may be due to the presence of false-positive signals. However, it must be noted that an observed dPCR bias could just as well be due to a technical error introduced while preparing the dilution series. In addition to this, quantitative bias may also be introduced by the underlying algorithm used by the digital PCR software to estimate the amount of input template from the concentration of positive to negative partitions. Recent data indicate that digital PCR partitions have small variations in size and this variation will always lead to an underestimation of the input amount of template when the algorithms fails to takes this variation into account^38^,^45^. Finally, the choice of the threshold may also induce a bias in the dPCR outcome, as a small number of partitions with intermediate fluorescence may be falsely classified as positive or negative and may therefore bias the quantitative outcome when chosen improperly^46^,^47^.

The patient sample quantifications were analyzed by Bland-Altman analysis. Bland-Altman analysis is a method for analyzing the agreement between two independent quantifications that is irrespective of “true” input quantity. Bland-Altman comparison of platform quantifications of patient-derived samples showed that the Quantstudio had the highest average difference with the other methods. Whereas the qPCR and the QX100 proved most similar in their average difference and minimal variation across all data, the false-positive partitions of the Quantstudio cause the observation of consistently higher abundances compared to the other two methods, which is especially obvious in the lower input range (Table 1, Fig. 2). The analysis of the limited dataset, from which samples with negative replicates were excluded, revealed that the non-constant variance observed in the complete dataset is likely caused by an increased variation in the low ranges due to sampling error. Hence, the true limits of agreement between the methods will better approach the limits estimated in the sub-analysis, compared to those estimated in the initial analysis that includes all samples.

The Bland-Altman analysis of the samples with higher copy numbers still revealed that the Quantstudio measured higher quantities than both the qPCR and the QX100 (Fig. 2). These results indicate that false-positive partitions impact Quantstudio quantification, both in the higher input ranges as well as in samples with low target abundance. Because of these false-positives, neither dPCR platform can provide an absolute certainty of the presence or absence of HIV DNA in samples with lowly abundant target. Although contamination cannot be excluded, we and others have shown that false-positive partitions can arise as artifacts in the QX100. The major drawback of the current dPCR platforms is that these single positive partitions cannot be isolated for subsequent analysis (e.g. sequencing) to confirm their artifactual nature or to indicate laboratory contamination. Classical qPCR samples on the other hand allow for post-PCR analysis, rendering this method superior to confirm presence or absence of HIV DNA in a given sample, regardless of quantification.

The present report indicates equally sensitive quantification of the QX100 compared to a semi-nested qPCR. This finding is contrary to earlier reports where dPCR was more sensitive compared to standard qPCR^26^, but in agreement with studies that also used a nested PCR set-up^11^,^25^. A properly designed nested PCR has a couple of advantages over direct PCR. It allows longer cycling and enrichment of rare target in a background of HIV-negative DNA, which effectively increases the limit of detection. Moreover, by using an additional primer sequence, the specificity of the assay is substantially enhanced, limiting the impact of non-specific amplification or laboratory contamination. A nested set-up with multiple primers may also be beneficial in dPCR, but the current platforms are unsuitable for the implementation of such a PCR set-up. However, this increased sequence specificity may not detect all sequence variants of HIV. DPCR may thus have an advantage over nested qPCR in the specific case of HIV, which is known to have multiple sequence variants between and within patients. Indeed, it has been shown that dPCR performs better than real-time PCR when small sequence variations are present in the priming regions^26^. Even single primer and probe mismatches will affect the accumulation of fluorescence and cause an underestimation in qPCR. In dPCR, mismatches will lead to droplets with a lower fluorescence intensity. However, as long as these droplets are above the threshold, they are assigned positive and will not affect quantification^26^,^47^. This higher tolerance to sequence variation may explain our observation that the dPCR platforms detected target in patient samples where the qPCR did not and our observation that the dPCR platforms detected higher target abundance than the qPCR. A comparison of multiple HIV templates from patients infected with different HIV subtypes should provide more insight into the performance of both assays on the quantification of HIV DNA variants *in vivo*.

In summary, the present findings show that dPCR enables direct and accurate quantification of HIV DNA. The QX100 proved equally sensitive as semi-nested qPCR, and superior by having less bias and higher precision, efficiency and quantitative linearity. However, due to the observation of false-positive signals with the current dPCR platforms, a semi-nested qPCR may still be preferred in a setup of low quantity detection to discriminate between absolute presence or absence of HIV DNA.

## Additional Information

**How to cite this article**: Bosman, K.J. *et al.* Comparison of digital PCR platforms and semi-nested qPCR as a tool to determine the size of the HIV reservoir. *Sci. Rep.* **5**, 13811; doi: 10.1038/srep13811 (2015).

## Supplementary Material

Supplementary Information

## Figures and Tables

**Table 1:**
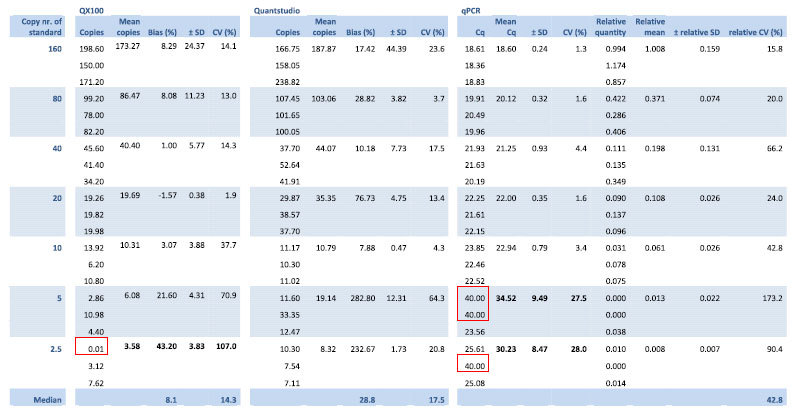
Dilution series output of the three methods.

The qPCR data is provided as Cq values and as transformed absolute values (relative to the standard curve) for comparison with the quantitative data of the dPCR platforms. Replicates for which no target could be detected were assigned a value of 0.01 in case of the digital platforms and a Cq of 40 in case of the qPCR and are indicated with red outlining. The resulting mean, bias, standard deviation and coefficient of variation of those samples are in bold to indicate that they have been influenced by the undetected replicates.
