# Supplementary material for: Comparison of digital PCR platforms and semi-nested qPCR as a tool to determine the size of the HIV reservoir
Source: Sci Rep. 2015 Sep 9;5:13811. doi: 10.1038/srep13811 (PMC4563360; doi:10.1038/srep13811)

Supplemental data to:

Comparison of digital PCR platforms and semi-nested qPCR as a tool to determine the size of the HIV reservoir.

KJ Bosman[1], M Nijhuis[1], PM van Ham[1], AMJ Wensing[1], K Vervisch[2], L Vandekerckhove[2], W De Spiegelaere[2]*

[1]Department of Medical Microbiology, Virology, University Medical Centre Utrecht, The Netherlands
[2]Department of Internal Medicine, HIV Translational Research Unit, University Ghent & University Hospital Ghent, Belgium

*Correspondence and requests for materials should be addressed to WDS (ward.despiegelaere@ugent.be).

Table S1. Water NTCs and PBMC DNA NTCs.

Type of control	QX100 Copies detected	Quantstudio Copies detected	Semi-nested qPCR Cq
**Water control (NTC)**	3.26	20.677	Undetermined
	3.75	29.232	Undetermined
		5.8435	
		10.3675	
		33.959	
		9.048	
		6.6265	
		8.9175	
**PBMC DNA NTC**	0	17.69	Undetermined
	0	7.0325	Undetermined
	0		Undetermined
	0		Undetermined
	0		Undetermined
	0		Undetermined

Table S2. Raw data patient samples. The qPCR data was transformed to absolute quantities by means of a standard curve. Replicates which returned an "undetermined" result were attributed a value of 0.01 copies. The data shown here is log-transformed.

Patient	Replicate	QX100	Quantstudio	Semi-nested qPCR
**1**	1	0.84	0.46	0.46
	2	0.77	0.90	0.84
	3	0.74	1.17	0.09
**2**	1	0.65	0.95	-2.00
	2	-2.00	0.83	-2.00
	3	-2.00	0.85	0.14
**3**	1	-0.05	1.00	0.19
	2	-2.00	0.75	0.59
	3	-2.00	1.08	0.06
**4**	1	1.13	0.76	1.09
	2	0.78	1.21	1.14
	3	0.97	0.92	0.63
**5**	1	0.35	0.28	-0.67
	2	0.36	0.00	-1.43
	3	0.17	0.20	-2.00
**6**	1	0.25	0.81	0.28
	2	0.56	1.14	0.71
	3	0.70	0.88	0.69
**7**	1	-0.08	0.82	-2.00
	2	-2.00	0.59	-2.00
	3	-2.00	0.70	-2.00
**8**	1	0.60	0.57	0.56
	2	0.67	0.94	0.16
	3	0.66	1.04	0.22
**9**	1	0.38	0.29	-2.00
	2	0.34	0.79	-2.00
	3	-0.16	0.97	-2.00
**10**	1	0.91	0.83	1.05
	2	0.85	1.00	1.17
	3	0.67	1.12	1.18
**11**	1	-2.00	0.21	0.20
	2	-2.00	0.55	-2.00
	3	-0.11	0.42	-2.00
**12**	1	1.47	1.57	1.67
	2	1.48	1.42	1.71
	3	1.41	1.49	1.62
**13**	1	0.16	0.52	-0.60
	2	-2.00	0.69	-0.06
	3	-0.11	0.86	-0.23
**14**	1	-2.00	1.09	-2.00
	2	-0.14	1.12	-2.00
	3	-0.09	0.72	-2.00
**15**	1	-2.00	1.00	-2.00
	2	-0.15	0.90	-2.00
	3	-2.00	0.93	-2.00
**16**	1	-2.00	0.21	-0.42
	2	-0.14	0.63	-2.00
	3	-0.15	0.55	-2.00
**17**	1	0.77	1.13	0.18
	2	0.72	1.04	0.78
	3	0.85	0.91	0.75
**18**	1	0.73	0.24	0.76
	2	0.18	1.16	0.80
	3	0.71	1.57	0.50
**19**	1	1.40	1.24	1.38
	2	1.28	1.40	1.26
	3	1.30	1.39	1.50
**20**	1	0.85	0.99	-2.00
	2	-0.11	0.55	0.34
	3	0.66	0.81	0.53

Table S3. Coefficient of variation (CV) calculated for the patients with positive quantifications in every replicate tested. Standard deviation is given over the three replicates, as well as the CV using the standard deviation as a percentage of the average log-transformed value.

Patient	Replicate	QX100			Quantstudio			Semi-nested qPCR		
		Value	SD	CV (%)	Value	SD	CV (%)	Value	SD	CV (%)
1	1	0.84			0.46			0.46		
	2	0.77			0.90			0.84		
	3	0.74	0.05	6.60	1.17	0.36	42.67	0.09	0.38	80.93
4	1	1.13			0.76			1.09		
	2	0.78			1.21			1.14		
	3	0.97	0.18	18.35	0.92	0.23	23.82	0.63	0.28	29.43
6	1	0.25			0.81			0.28		
	2	0.56			1.14			0.71		
	3	0.70	0.23	46.28	0.88	0.17	18.19	0.69	0.24	43.29
8	1	0.60			0.57			0.56		
	2	0.67			0.94			0.16		
	3	0.66	0.04	6.33	1.04	0.25	29.33	0.22	0.21	67.01
10	1	0.91			0.83			1.05		
	2	0.85			1.00			1.17		
	3	0.67	0.13	15.66	1.12	0.15	14.87	1.18	0.07	6.54
12	1	1.47			1.57			1.67		
	2	1.48			1.42			1.71		
	3	1.41	0.04	2.54	1.49	0.07	5.00	1.62	0.04	2.68
17	1	0.77			1.13			0.18		
	2	0.72			1.04			0.78		
	3	0.85	0.06	8.21	0.91	0.11	10.91	0.75	0.34	59.94
18	1	0.73			0.24			0.76		
	2	0.18			1.16			0.80		
	3	0.71	0.31	58.19	1.57	0.68	68.47	0.50	0.16	23.38
19	1	1.40			1.24			1.38		
	2	1.28			1.40			1.26		
	3	1.30	0.07	5.10	1.39	0.09	6.74	1.50	0.12	8.80
Average CV:				18.58			24.45			35.78
Median CV:				8.21			18.19			29.43

Figure S1. Residual regression for all patients and patients with high input quantities. Regression on the absolute residuals is calculated for all samples (D, E, F) with their corresponding mean difference (solid blue lines in A, B, C) and for only the high input subset to the adjusted mean difference (G, H, I, absolute residuals to solid red line in A, B, C).

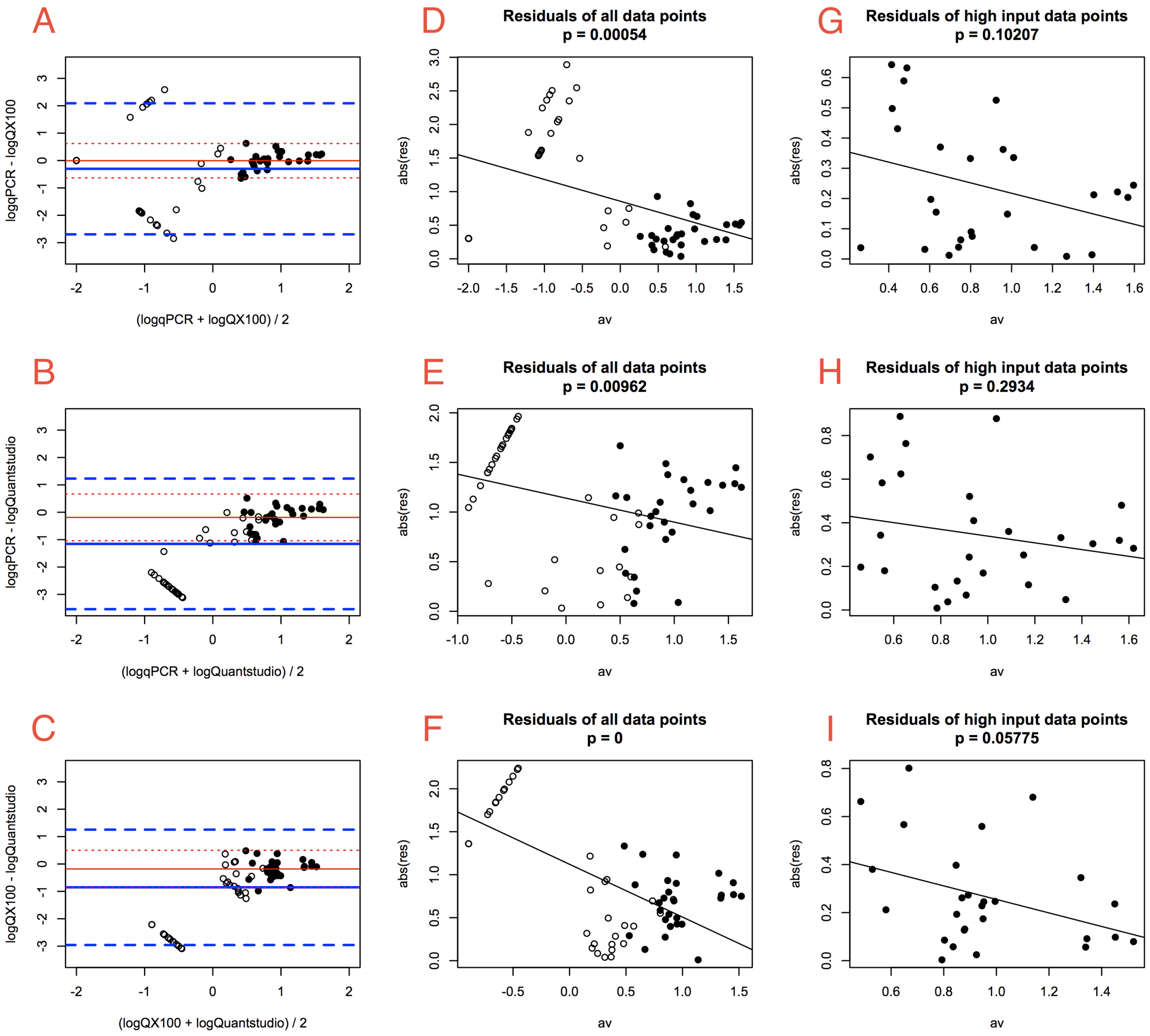

Supplement: Supplementary Information [file srep13811-s1.pdf]
